# Theoretical Evaluation of Novel Thermolysin Inhibitors from *Bacillus thermoproteolyticus*. Possible Antibacterial Agents

**DOI:** 10.3390/molecules26020386

**Published:** 2021-01-13

**Authors:** Emilio Lamazares, Desmond MacLeod-Carey, Fernando P. Miranda, Karel Mena-Ulecia

**Affiliations:** 1 Pathophysiology Department, Biotechnology and Biopharmaceutical Laboratory, School of Biological Sciences, Universidad de Concepción, Victor Lamas 1290, P.O. Box 160-C, Concepción 4079386, Chile; elamazares@udec.cl; 2 Inorganic Chemistry and Molecular Materials Center, Instituto de Ciencias Químicas Aplicadas, Facultad de Ingeniería, Universidad Autónoma de Chile, El Llano Subercaseaux 2801, San Miguel, Santiago 8900000, Chile; desmond.macleod@uautonoma.cl; 3 Instituto de Fisiología, Facultad de Medicina, Universidad Austral de Chile, Valdivia 5090000, Chile; fmiranda959@gmail.com; 4 Departamento de Ciencias Biológicas y Químicas, Facultad de Recursos Naturales, Universidad Católica de Temuco, Ave. Rudecindo Ortega 02950, Temuco 4780000, Chile; 5 Núcleo de Investigación en Bioproductos y Materiales Avanzados (BIOMA), Facultad de Ingeniería, Universidad Católica de Temuco, Ave. Rudecindo Ortega 02950, Temuco 4780000, Chile

**Keywords:** thermolysin, antibacterial agents, docking, molecular dynamics, MM-PBSA, ADME-Tox

## Abstract

The search for new antibacterial agents that could decrease bacterial resistance is a subject in continuous development. Gram-negative and Gram-positive bacteria possess a group of metalloproteins belonging to the MEROPS peptidase (M4) family, which is the main virulence factor of these bacteria. In this work, we used the previous results of a computational biochemistry protocol of a series of ligands designed in silico using thermolysin as a model for the search of antihypertensive agents. Here, thermolysin from Bacillus thermoproteolyticus, a metalloprotein of the M4 family, was used to determine the most promising candidate as an antibacterial agent. Our results from docking, molecular dynamics simulation, molecular mechanics Poisson–Boltzmann (MM-PBSA) method, ligand efficiency, and ADME-Tox properties (Absorption, Distribution, Metabolism, Excretion, and Toxicity) indicate that the designed ligands were adequately oriented in the thermolysin active site. The Lig783, Lig2177, and Lig3444 compounds showed the best dynamic behavior; however, from the ADME-Tox calculated properties, Lig783 was selected as the unique antibacterial agent candidate amongst the designed ligands.

## 1. Introduction

The increase in bacterial diseases worldwide has been relevant in recent years [1]. It is proposed that more than 117 conditions of bacterial origins will be described worldwide in 2020–21. According to the World Health Organization (WHO), based on bacterial infections, the highest priority is to research and develop antibacterial drugs against Gram-positive and Gram-negative bacteria [2].

Bacteria of the Vibrio, Legionella, Clostridium, Listeria, Staphylococcus, and Pseudomonas genus are keys factors of many diseases such as cholera [3,4], ulcerative gastritis [5], and gastric carcinoma [6], affecting millions of people around the world. These bacteria have a significant group of common proteins that are crucial factors in the pathogenesis of several diseases [7]. This group of proteins is named the MEROPS peptidase family (M4 family) [8].

An M4 family is an essential group of metalloproteins that has close similarities between them [9]. This group is also commonly called the Thermolysin-Like-Proteinase (TLPsa) family [9]. This name is assigned due to the high similarity with thermolysin since they share a common consensus sequence (HExxH) [7], which is part of the catalytic domain together with Zn^2+^. Therefore, the search for effective inhibitors of the M4 family proteins including thermolysin is one of the novel strategies in the design of new third and fourth generation antibiotics [10].

**Figure 1:**
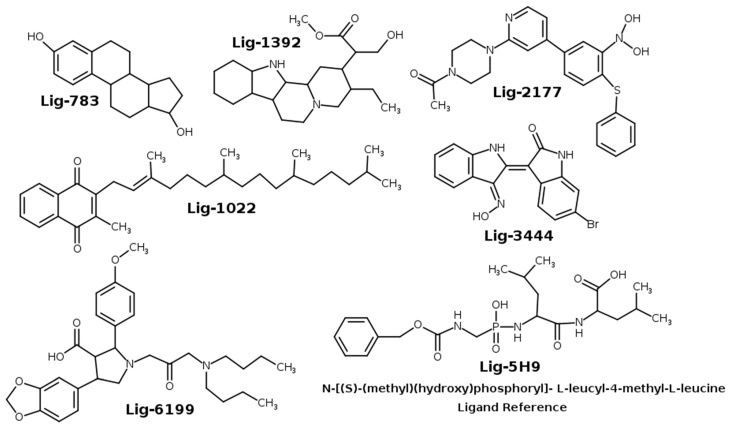
Molecular structure of thermolysin inhibitors evaluated as possible antibacterial agents.

Thermolysin is secreted by *Bacillus thermoproteolyticus*. It is considered a metalloproteinase containing Zn^2+^ in its active site, which is an essential cofactor for the enzyme catalytic activity [7]. This protein was one of the first metalloproteins to be isolated. Its crystallographic structure was resolved a long time ago, as a result, it is widely used as a disease studies model [11,12]. Our research group has previously designed new thermolysin inhibitors as possible antihypertensive drugs by using a combination of QSARIN and virtual screening methods [11]. Six compounds were selected from that previous study (Figure 1) and were evaluated as possible antibacterial agents. We present a computational biochemistry protocol based on docking, molecular dynamics simulation, molecular mechanics Poisson–Boltzmann (MM-PBSA) method, ADME-Tox properties (Absorption, Distribution, Metabolism, Excretion, and Toxicity) and ligand efficiency methodologies to comprehensively evaluate these molecules as possible antibacterial agents.

## 2. Results and Discussion

In a recent work [11], we obtained 133 compounds with described experimental inhibitory activity against thermolysin from international literature reports. To design a new and more efficient inhibitor ligand for thermolysin, we performed a combination of QSARINS and virtual screening technique methods and determined the molecular fragments that interacted with the thermolysin active site. Based on these results, we created new ligands, whose structure is presented in Figure 1. In this work, we employed a computational protocol to verify whether the designed compounds could be considered as good candidates for use as antibacterial agents.

### 2.1. Molecular Docking

We used the docking experiments to determine the position and binding mode of a protein–ligand complex with minimum energy due to their usefulness in the drug’s design [11,12,13,14]. In this work, docking experiments were used to determine how the ligands designed in silico presented in Figure 1 were positioned in the thermolysin active site. The crystallographic structure of thermolysin was obtained from the Protein Data Bank (PDB id:5DPF).

**Figure 2:**
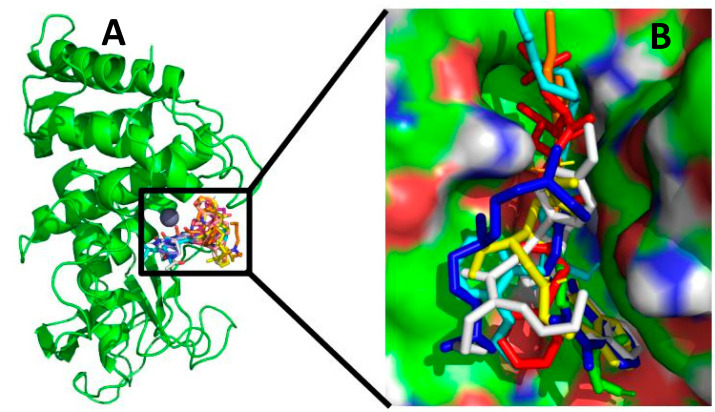
Alignment of the best poses of ligands designed in silico compared to the Lig5H9 reference compound into the thermolysin active site. (**A**) The thermolysin protein in green, the sphere represents the Zn^2+^ ion at the thermolysin active site. (**B**) A close-up of the ligands’ best poses in the active site. In red: the reference ligand Lig5H9, green: the Lig783, blue: the Lig1022, yellow: the Lig1392, cyan: the Lig2177, orange: the Lig3444, and white: the Lig6199.

As shown in Figure 2, the docking poses obtained were adjusted acceptably with the available inhibitory x-ray crystal structures. All ligands were adequately located at the thermolysin pocket. According to the docking experiments, the best poses were computed, and, as shown in Table 1, 86% of the poses obtained had a ΔG_binding_ energy greater than 7 kcal/mol, and 43% had a root-mean-square deviation (RMSD) below 2 Å. The most stable complex was Lig5H9-5DPF, with the most negative free binding energy (−8.2 kcal/mol) and the second-lowest RMSD value (Table 1) out of all the ligand–protein complexes analyzed (0.90 Å). The RMSD values were obtained by comparing the best poses of the ligand-protein complexes with the crystallographic structure from the Protein Data Bank (PDB id:5DPF). The reference ligand 5H9 was re-docked using the same methodology as other ligands to validate our docking experiments.

**Figure 3:**
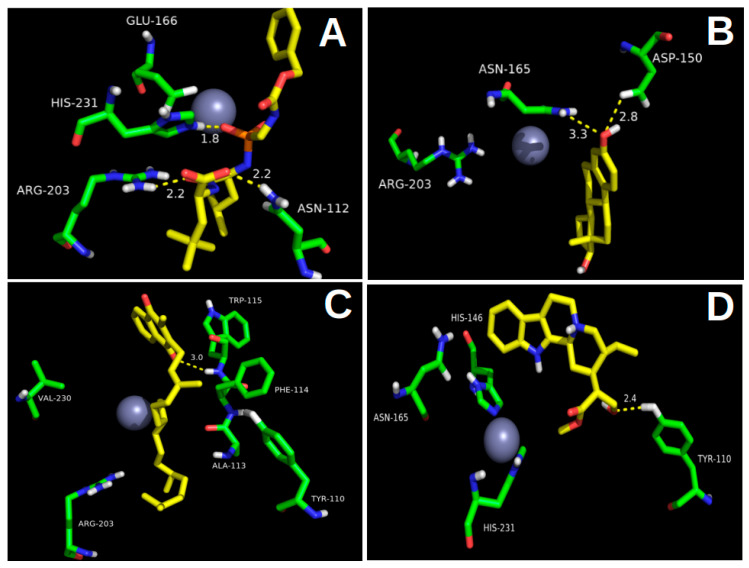

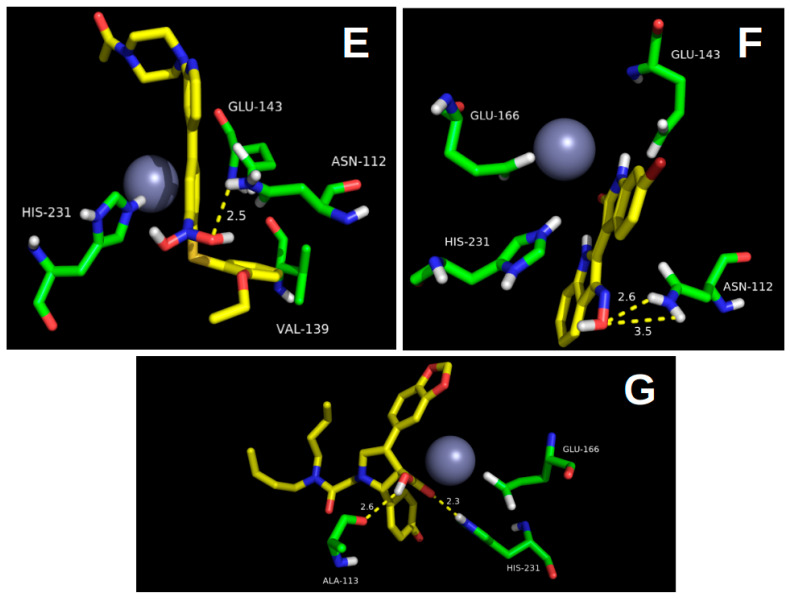
Binding modes’ structural details of the ligands designed in silico placed into the thermolysin active site: (**A**) Lig5H9 reference compound, (**B**) Lig783, (**C**) Lig1022, (**D**) Lig1392, (**E**) Lig2177, (**F**) Lig3444, and (**G**) Lig-6199. The right side corresponds to the 2D representation of the ligand’s interactions studied in the thermolysin active site. The grey spheres represent the Zn^2+^ ion in the thermolysin active site.

According to the docking experiment results, Lig5H9 possesses a *POOH* group, which interacts via the H-bond with the His231 residue. The carboxyl and the closest amide groups present two hydrogen-bonding interactions with Asn112 and Arg103, respectively. These interactions were also found to be present in the thermolysin crystal structure (Figure 3A). However, the hydrophobic ring of Lig5H9 was oriented toward a hydrophobic pocket formed by the Ala113, Phe114, Trp115, and Tyr157 in the thermolysin active center. These interactions were not present in the crystal structure.

From all the molecules designed in silico, the best oriented in the thermolysin pocket was Lig3444. The Lig3444-5DPF complex had the second most negative binding energy (below the reference ligand 5H9) with −8.1 kcal/mol, followed by Lig783 and Lig2177 (ΔG_binding_ = −8.0 kcal/mol), see Table 1.

Another stable complex obtained from the docking experiments was the Lig3444-5DPF. The Lig3444 ligand possesses an oxime group, which interacts by hydrogen bonding with Asn112. The pyrrolidine ring had an H-bond interaction with Glu143, which provides high stability to this compound in the active pocket of thermolysin (Figure 3F).

The third and fourth most negative binding free energies (ΔG_binding_ of −8.0 kcal/mol) corresponded to the Lig783 and Lig2177 ligands. The Lig783-5DPF complex stability is explained by the hydrogen bond interaction between the phenolic hydroxyl group with Asp150 and Asn165. The hydrocarbon skeleton was oriented into a hydrophobic pocket formed by Phe114, Trp115, and Trp157 (Figure 3B). Lig2177 had an *N*-hydroxy-hydroxylamine group, which interacted with Asn112 forming an H-bond. The terminal phenyl ring was located in a hydrophobic pocket formed by the Phe130, Leu133, and Val139, which provides high stability to this complex (Figure 3E).

These are the most stable ligand–protein complexes determined by the docking experiments. To analyze the stability of these interactions over time, we carried out molecular dynamics simulations as described below.

### 2.2. Molecular Dynamics Simulation

Molecular dynamics simulations provide trajectories with structural information at the molecular level [15,16,17]. Our main goal was to determine if the interactions encountered in the docking experiments were maintained over time. In this sense, we performed an integral data analysis obtained from the molecular dynamics simulations results considering the RMSD, RMSF, H-bond interaction stability, and radius of gyration across the time of simulations.

#### 2.2.1. Root-Mean-Square Deviation (RMSD) Parameter

**Figure 4:**
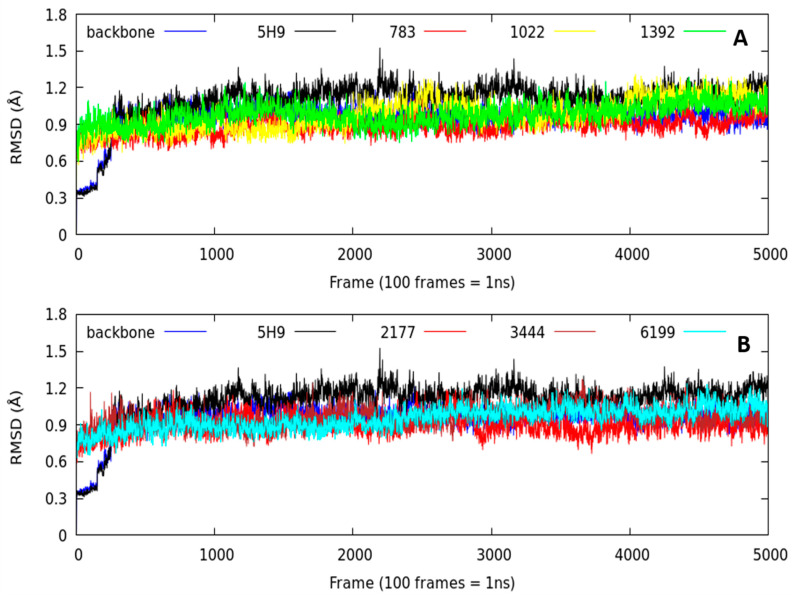
Comparative plots of RMSD values against simulation time corresponding to the molecular dynamics of the backbone, 5H9 ligand reference, and molecules designed in silico-thermolysin complexes. (**A**) is the comparison between backbone, 5H9, Lig783, Lig1022, Lig1392; (**B**) is the comparison between backbone, 5H9, Lig2177, Lig3444, Lig6199.

As a stability criterion, we analyzed the RMSD (root-mean-square deviation) parameter (RMSD < 2.0 Å) [18,19,20]. This descriptor gives us an idea of how the systems evolved during the 50 ns of simulation time. From the first 3 ns, all the ligand–protein complexes studied remained stable over time with a minimal variation (Figure 4). All the systems had RMSD values lower than 1.4 Å, even below the Lig5H9-5DPF complex (our reference system), indicating that the compounds designed in silico remained stable over time into the thermolysin pocket.

The averaged RMSD parameter is another stability criterion for the studied systems (Table 2). We observed no significant differences in the RMSD averaged value over time. The most stable complex was Lig2177-5DPF (0.90 ± 0.07 Å). This system had the lowest RMSD value of the complexes studied, even lower than our reference system (Lig5H9-5DPF), which had an average RMSD value of 1.11 ± 0.13 Å.

It is important to note that the Lig2177-5DPF complex presented one of the most negative binding energies in the docking experiments (Table 1). The Lig783-5DPF, Lig6199-5DPF, and Lig3444-5DPF complexes had RMSD values close to the Lig2177-5DPF complex (0.93 ± 0.08 Å, 0.97 ± 0.05 Å and 0.98 ± 0.07 Å, respectively). Except for Lig6199-5DPF, the Lig783-5DPF and Lig3444-5DPF systems had similar binding energies than the Lig2177-5DPF complex docking experiments.

The least stable complexes among the series (does not mean that they were unstable) were Lig1022-5DPF, Lig1392-5DPF, and our reference system was Lig5H9-5DPF with RMSD values of 1.01 ± 0.11 Å, 1.10 ± 0.15 Å, and 1.11 ± 0.13 Å, respectively. The RMSD standard deviation value was low, indicating that the RMSD values remained constant with small fluctuations over time (50 ns). The simulation time studied was sufficiently long to determine the ligand binding mode in the thermolysin active center. We can conclude that the RMSD parameter did not detect significant variations in the dynamic behavior of the complexes studied. To determine which interactions were responsible for this behavior, it is necessary to analyze the hydrogen bond number and stability details at the molecular level across the time of MD simulations.

#### 2.2.2. Hydrogen Bond Interactions (H-Bond)

**Figure 5:**
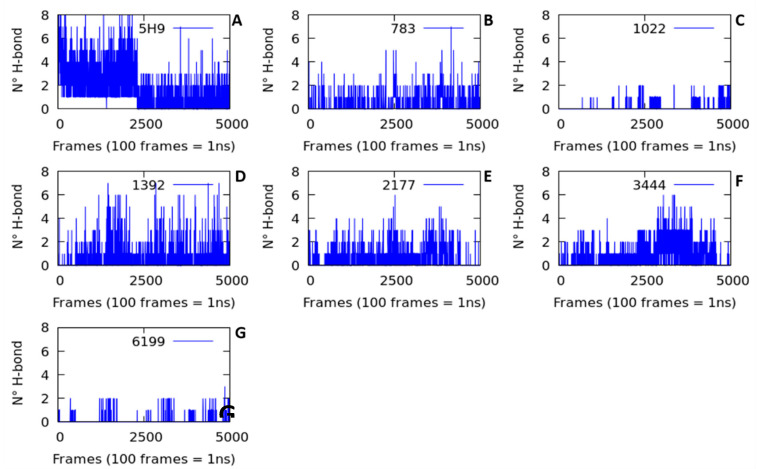
The number of hydrogen bonds between the thermolysin and the ligands designed in silico during 50 ns of molecular dynamics simulation. (**A**) Lig5H9 reference compound, (**B**) Lig783, (**C**) Lig1022, (**D**) Lig1392, (**E**) Lig2177, (**F**) Lig3444, and (**G**) Lig-6199.

The number and occupancies of H-bond interactions during the 50 ns of simulation time were quantified to determine the studied complex stability. The number of H-bond interactions was low in all the ligand-protein complexes studied (Figure 5). All systems had an H-bond average lower than 1.5 (Table 2), indicating that these interactions do not significantly influence the stability of the ligands in the thermolysin active site.

In addition to the low number of H-bonds, interaction stability was also low. The standard deviation of H-bond interactions for all systems was higher than the population average, indicating the low stability of these interactions (Table 2). Another interesting fact that must be emphasized is the H-bond interaction occupancies over time. The occupancy term (%) refers to when the H-bond interaction is maintained at less than 3 Å of distance during the 50 ns of simulation. More than 50% occupancy was taken as a stability criterion [21,22].

The highest occupancy (24.63%) was obtained from the hydrogen bond interaction formed by *Lig5H9-OH···O = Glu166* maintained at an average distance of 1.75 ± 2.38 Å during the molecular dynamics simulation time (50 ns). The second-highest occupancy (20.36%) was found for the H-bond interaction formed by *Lig783-OH···O = Asp150*, with 2.83 ± 1.60 Å of average distance during the trajectory.

From this analysis, the low number of hydrogen bonds and the instability of the interactions cannot explain the complex stability shown in the molecular dynamics simulations by using the H-bond parameter. Thus, it was necessary to analyze other parameters such as radius of gyration, the results of which are presented below.

#### 2.2.3. Radius of Gyration (Rg)

The ligand-protein complexes stability already analyzed using the RMSD and H-bond interaction parameters cannot be completely described using these two parameters. We examined the compaction degree of the ligand-5DPF complexes during 50 ns of molecular dynamics trajectories. The compaction degree of the complexes studied is described using the radius of gyration (Rg) parameter [23,24,25]. This variable is defined as the root mean square distance of the mass of an atom’s collection from a common mass center. There are two analysis levels in the Rg graph: the value of the parameter over time and its fluctuation [23,24,25].

**Figure 6:**
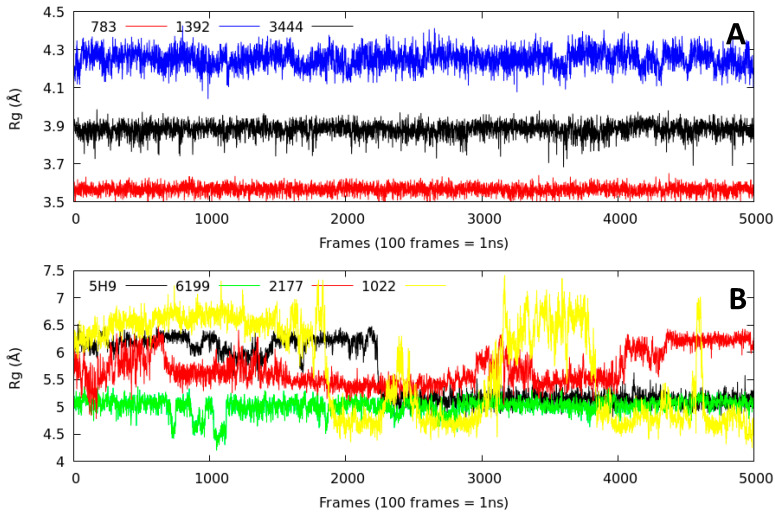
The radius of gyration (Rg) of Cα atoms in the ligand–5DPF complexes during 50 ns of molecular dynamics simulation. (**A**) is the comparison between Lig783, Lig1392 and Lig3444. (**B**) is the comparison between Lig5H9, our reference compound, Lig6199, Lig2177 and Lig1022.

The Lig783-5DPF, Lig3444-5DPF, and Lig1392-5DPF complexes were more compact over time, as presented in Figure 6. These systems exhibited the lowest RMSD values among all the series (Table 2) and remained stable over time (50 ns) without appreciable conformational changes. Another compact complex obtained was Lig6199-5DPF, although the Rg value was slightly higher than Lig783-5DPF, Lig3444-5DPF, and Lig1392-5DPF.

The trajectory behavior observed for the Lig5H9-5DPF, Lig1022-5DPF, and Lig2177-5DPF complexes were different from the previously described ones (Figure 6). The Lig5H9-5DPF complex presented few conformational fluctuations during the first 22 ns of trajectory. From this time, the system stabilizes until the end of the trajectory (Figure 6).

**Figure 7:**
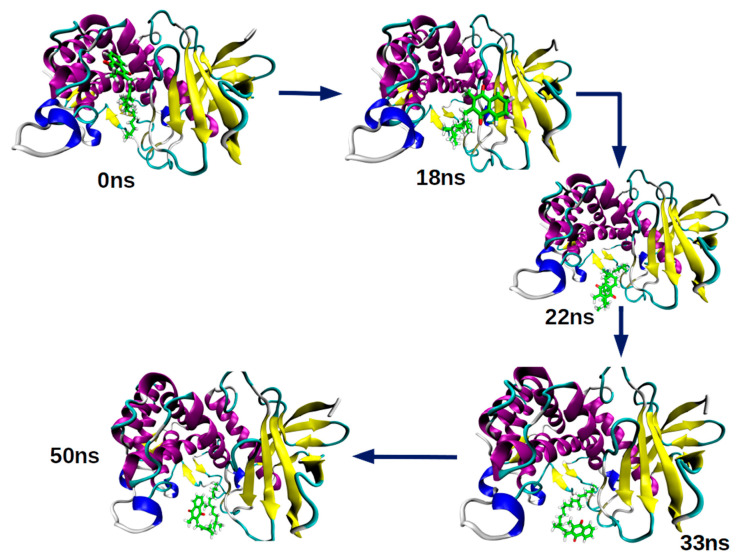
The Lig1022-5DPF complex’s conformational changes during the molecular dynamics simulation time (50 ns).

The Lig1022-5DPF complex was the least compact of all systems studied based on the fluctuations in the first 18 ns of simulation time (Figure 6). A conformational change (Figure 7) was observed at this time with an Rg value that decreased abruptly. Then, its value increased again from 33 ns, demonstrating the low stability of this complex.

#### 2.2.4. Root-Mean-Square Fluctuation (RMSF) Parameter

The root-mean-square fluctuation (RMSF) parameter measures the different amino acid residues’ flexibility of the thermolysin structure during the 50 ns trajectory [26]. Higher RMSF values represent greater flexibility of movement, while lower RMSF values suggest the existence of mobility restrictions during the simulation.

**Figure 8:**
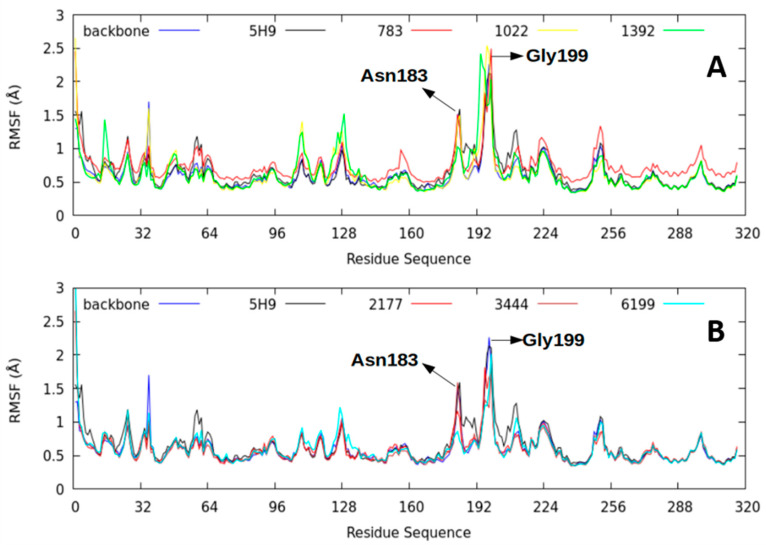
The ligand-thermolysin complexes’ backbone atoms’ root mean square fluctuation of molecular dynamics simulations (50 ns) at 298.15 K. (**A**) is the comparison between the Thermolysin backbone and the ligands Lig5H9, our reference compound, Lig783, Lig1022 and Lig1392. (**B**) is the comparison between the Thermolysin backbone and the ligands Lig5H9, our reference compound, Lig2177, Lig3444 and Lig6199.

The lowest RMSF values were found between amino acids 129–155 and 161–165 in all the ligand–protein complexes studied (Figure 8). The area covered by these amino acids was in contact with the studied ligands and remained more rigid than the 5H9 reference. Specifically, this area corresponded to the thermolysin active site and the consensus sequence (HExxH) within the M4 family proteins [9,27]. In this study, the sequence was His142-Glu166-Tyr157-His146-His231 for the 5DPF protein. The most flexible amino acid residues were Asn183 and Glu199 because of their structural role rather than a catalytic one.

### 2.3. Molecular Mechanics Poisson-Boltzmann Surface Area (MM-PBSA)

The molecular mechanics Poisson-Boltzmann surface area (MM-PBSA) method is a free energy decomposition analysis at the molecular level that explores which energy components contribute favorably or adversely to the stability of ligand-protein complexes [28].

Table 3 shows that the Van der Waals electrostatic and the nonpolar solvation terms contributed to the stability of the studied complexes. However, the ∆G_polar_ term is destabilizing in these systems (Table 3).

The Lig5H9-5DPF complex (our reference ligand) was the most stable with the most negative ∆G_binding_ value, which agreed with the results of the docking experiments.

The Lig1022-5DPF complex showed the most negative binding energy from all ligands designed in silico. This complex had the third most negative binding energy in docking experiments (Table 1), and according to the MM-PBSA calculations results, the Lig1022-5DPF complex was mainly stabilized via Van der Waals interactions.

The Lig6199 and Lig1392 complexes showed the second and third most negative ∆G_binding_ in the MM-PBSA calculations (Table 3). These systems presented the lowest negative energies in the docking experiments due to the loss of stabilizing interactions during the molecular dynamics simulations. In addition to the Lig1022-5DPF complex, these two systems were also stabilized through Van der Waals interactions.

**Figure 9:**
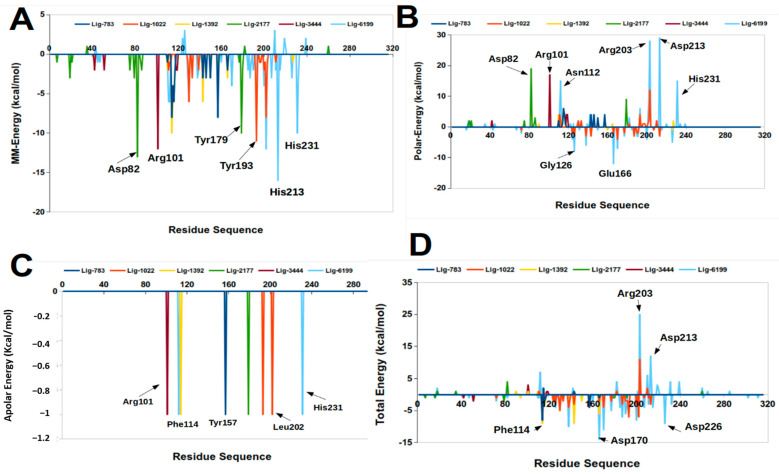
Decomposition of the binding free energy per residue on the thermolysin pocket. (**A**) represent the MM-Energy contribution. (**B**) represent the polar energy contribution. (**C**) represent the apolar energy contribution and (**D**) represent the total energy contribution.

The detailed analysis by residue (Figure 9) showed that the Phe126, Asp138, Glu166, Glu187, and Asp170 residues contributed favorably to the complex ∆G_binding_. The Glu166 residue is present in the active center conserved sequence of M4 family proteins [9,27] and is essential for the catalytic activity [9,27]. This result was corroborated by our molecular dynamics simulations, together with free energy calculations.

The Asp82, Arg101, Asn112, Phe114, and His231 amino acids contributed favorably to the electrostatic origin and Van der Waals interactions. His231 is another essential residue located in the thermolysin active center consensus sequence [9,27].

### 2.4. Ligand Efficiency Calculation and Absorption, Distribution, Metabolism, Excretion, and Toxicity (ADME-Tox) Properties

At this point, we have analyzed the ligand–thermolysin complexes’ molecular stability. In the next section, we analyze the affinity of compounds designed with thermolysin and the ADME-Tox properties. These results will resolve which of the designed compounds are the best candidates for antibacterial agents, thus minimizing the risk as much as possible.

We used four parameters in this analysis, the dissociation constant (K_d_), ligand efficiency index (LE), binding efficiency index (BEI), and lipophilic ligand efficiency (LLE) to compare and select the best candidates for antibacterial agents. The dissociation constant (K_d_) parameter measures the strength of the ligand–protein interaction. Low Kd values indicate a strong interaction between the ligand and the protein. The dissociation constant was calculated using Equation (4). Lig5H9 showed the most robust interaction with significant differences between the rest of the ligands studied (Table 4). The three designed compounds, Lig3444, Lig783, and Lig2177, had the lowest K_d_ values, suggesting that they could be good candidates for antibacterial agents.

The ligand efficiency index (LE) correlates the ligand–protein binding energy with the compound number of atoms without considering the hydrogens in its structure [29]. In our work, for an adequate drug candidate, we considered LE > 0.3 kcal/mol HAC as a reference [29,30,31]. Consistent with the reference value, compounds like Lig783 and Lig3444 are good candidates as thermolysin inhibitors because they had LE values of 0.40 and 0.36, respectively (Table 4). Studies on common drugs have given LE values between 0.5 and 0.52 for oral administration drugs [29]. We suggest that the Lig783 and Lig3444 compounds could be good candidates for orally administered drugs.

The binding efficiency index (BEI) relates the dissociation constant (K_d_) of the ligand and its molecular weight [32]. As reference values, we considered a rate of 20 < BEI < 27. These values were obtained from known drugs such as bortezomid (BEI = 21), a commercially available proteasome inhibitor [33]. The BEI values for our designed ligands were lowest than the reference values; only Lig783 showed a BEI value similar to bortezomib (Table 4) [33].

**Figure 10:**
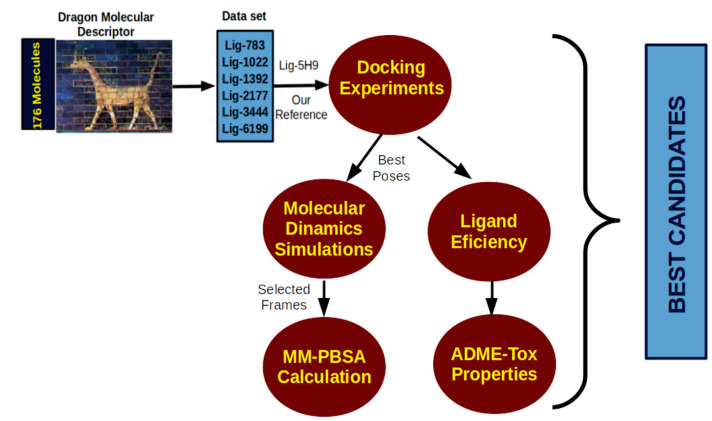
Computational protocol for the evaluation of possible antibacterial agents.

Another essential variable to consider is the lipophilic ligand efficiency (LLE). This parameter measures the ligand-binding capacity with the protein and its lipophilic power [29]. In our case, the LLE’s reference values were between 5–7 units; these values were calculated based on oral administration of known drugs [34]. The calculated LLE values for our ligands designed in silico were lower than the reference values, mainly because the cLogP variable had relatively high values, which indicates the high lipophilic power for these compounds (Table 4). Suppose we focus on analyzing the molecular chemical structure (Figure 10), we can observe the apolar hydrocarbon skeletons that could influence our lipophilic ligand capacity.

The pharmacokinetic prediction for absorption, distribution, metabolism, and elimination (ADME) of a drug is necessary to be known before suggesting a promising drug candidate molecule. Additionally, some toxicological parameters (Tox) [35,36,37,38] are critical to know. In this work, we calculated several parameters like the molecular weight (MW), the octanol/water partition coefficient (cLogP), hydrogen bond acceptor count (HBA), hydrogen bond donor count (HDA), and rotatable bond count (RB). We used the SwissADME web server [39]. As a toxicological criterion, these results were compared with the Lipinski [40], Veber [41], and Pfizer [42] rules. Thus, if any compounds designed in silico comply with only one Lipinski rule, this molecule is not the right candidate. In contrast, according to the Veber and Pfizer rules, a right ligand candidate must agree with both rules.

Our reference, ligand 5H9 met all the parameters of the Lipinski rule (Table 4). However, it did not meet any of Veber rule parameters. Ligand 5H9 only agreed with one of the Pfizer rule parameters, indicating that this compound could have high oral availability. However, its high lipophilic power could have some toxicological potential. The Lig783, Lig1392, and Lig3444 compounds complied with all of the Lipinski, Veber, and Pfizer rulers. Thus, we can consider these ligands as the best antibacterial candidate agents from our analysis. The Lig1022, Lig2177, and Lig6199 molecules presented a high lipophilic power, resulting in being inadequate candidates as this parameter suggests some toxicological consequences.

## 3. Computational Protocol

We planned a computational biochemistry protocol to evaluate thermolysin inhibitors as possible antibacterial agents, as shown in Figure 10.

The molecules that we studied in this work were previously designed by our research group using the QSAR-Insubria (QSARIN) method [11]. The molecular structures of these possible thermolysin inhibitors were sketched using Avogadro software version 1.2.0 [43]. The optimized geometries were obtained through Density Functional Theory (DFT) calculations using the B3LYP/ma-def2-SVP basis set implemented in Orca 4.2.1 software package [44,45]. The full optimized geometries were checked by their imaginary frequencies counting. The thermolysin inhibitors’ molecular structures studied are represented in Figure 1. To examine the compound’s interaction in the thermolysin pocket, we used the docking experiment to obtain each molecule’s optimized geometries.

### 3.1. Docking Procedure

The full optimized geometries for the ligands obtained from quantum calculation were used in docking experiments. The molecules were prepared at pH = 7.4 using Autodock Tools [46]. The thermolysin x-ray crystallography structure was obtained from Protein Data Bank (PDB) [47] with PDBid: 5DPF (resolved at 1.47 Å) [48]. This protein was prepared by the addition of all hydrogen atoms at pH = 7.4. The water molecules around the protein were eliminated. The size of the grid box was 16.875 × 14.14 × 16.875 Å^3^ around the mass centers of our reference ligands (5H9) discharged from the x-ray crystallography structure from PDB whose coordinates were x = 11.844 y = −40.829, and z = 64.20. The Zn^2+^ ion was used in all docking experiments because it is present in the thermolysin M4 family pocket.

In all docking procedures, we used a grid spacing of 0.375 Å, the number of modes was 10, and the energy rank was set up to 1 kcal/mol. The correct docking results were analyzed by re-docking the 5H9 reference ligand under the same docking protocol of the other compounds using Autodock Vina software version 1.1.2 [49,50]. The best docking poses were selected using binding energy (kcal/mol) and the positional root-mean-square deviation (RMSD) [51].

The best energetically good poses and lowest root-mean-square deviation of the complex were selected for molecular dynamics simulations and ligand efficiency calculations. The reproducibility of the docking results was verified by calculating the root-mean-square deviation (RMSD) between the possible antibacterial compounds. The 5H9 ligand crystallographic structure from the Protein Data Bank was used as a reference. These calculations were performed by the LigRMSD server 1.0 program [52]. All docking figures were made using Pymol software version 1.8 [53].

### 3.2. Molecular Dynamics Simulation

The best conformational poses for each ligand–thermolysin complex from the docking experiments were obtained as the start point for the molecular dynamics simulations. Each complex was placed into a water box of 15 × 15 × 15 Å^3^ using the TIP3P water model [54,55]. Topologies and parameters of the ligands were obtained by the SwissParam web Server [56]. All molecular dynamics simulations were described using CHARMM36 and CGenFF force field for the thermolysin and the possible antibacterial compounds [57,58,59,60,61].

The ligand–thermolysin complexes were submitted to 50,000 steps for energy minimization using the conjugated gradient methodology to reduce any close contact. The working temperature was 298.15 K using the weak coupling algorithm [62]. The Van der Waals cutoff was fixed to 12 Å, and we applied a backbone constraint to all complexes by the NPT (Number of particles, Pressure and Temperature constant) ensemble. Considering the long ranges of the electrostatic forces, we used the Particle Mesh Ewald (PME) approach [63]. The velocity Verlet algorithm with a 1.0 fs time step was used to solve the motion equations. All complexes were submitted to 2.0 ns of equilibration and 50 ns of molecular dynamics simulation using the NAMD 2.13 software package [64]. The trajectories’ analysis and scripts were performed by VMD software version 1.9.3 [65].

### 3.3. Free Energy Calculations

This work’s computational protocol combined molecular dynamics simulation and MM-PBSA to study the ligands and thermolysin interactions. The binding free energy was calculated using *g_mmpbsa* package version 5.1.2 [66], and a Gromacs tool to compute the ligand-free binding energy [66]. Gromacs is an implicit solvent method to obtain the different energy between the ligand–thermolysin complexes.

We extracted the last 300 frames from the 50 ns of molecular dynamics simulation to calculate each complex binding free energy. Therefore, the MM-PBSA method calculates the free energy decomposition into contributions. The free energy of the ligand–thermolysin complexes was calculated according to the following equation:

(1) $${\Delta G}_{\mathrm{binding}}{\;=\;G}_{\mathrm{complex}}-\left(G_{\mathrm{Thermolysin}}{+G}_{\mathrm{ligand}}\right)$$

In Equation (1), G_complex_ corresponds to the thermolysin–ligand complex energy, G_Thermolysin_, and G_ligand_ is the protein and ligand energy. The following equation was used to calculate the protein, ligand, and complex free energy separately.

(2) $$G_{x}{\;=\;E}_{\mathrm{bond}}{\;+\;E}_{\mathrm{vdw}}{\;+\;E}_{\mathrm{elect}}{\;+\;G}_{\mathrm{polar}}{\;+\;G}_{\mathrm{Apolar}}$$

In Equation (2), G*_x_* can be G_complex_, G_Thermolysin,_ or G_ligand_. The E_bond_ represents the interactions that include bond, angle, and dihedral angle; E_elect_ is the electrostatic energy contribution; and E_vdw_ is a Van der Waals energy contribution. The G_polar_ represents the polar free energy contribution, which was calculated using the continuum solvent Poisson-Boltzmann (PB) model included in the APBS (Adaptive Poisson-Boltzmann Solver) software version 1.4.1 [67]. The nonpolar free energy contribution was calculated according to the following equation:

(3) $$G_{\mathrm{Apolar}}\;=\;\gamma \mathrm{SASA}\;+\;\beta$$

where γ represents the coefficient related to the solvent-surface tension, which in our case had a 0.0072 kcal/mol/Å^2^ value; SASA is the solvent-accessible surface area with a 1.4 Å; and β is a fitting parameter.

We also decomposed the overall binding energy per residue because we needed to know every amino acid contribution. These contributions were calculated using python script MmPbSaStat.py, and the amino acid’s individual contribution in binding was calculated by MmPbSaDecomp.py scripts [66].

### 3.4. Ligand Efficiency Calculations

Ligand efficiency metrics is a series of parameters that measure the relationship between the binding energy and the molecule size [29]. These parameters significantly predict how efficient a compound will be as a possible drug [29,31,68,69]. In our case, the ligand efficiency calculations were performed through several parameters such as the dissociation constant (K_d_), the ligand efficiency index (LE), the binding efficiency index (BEI), and the lipophilic ligand efficiency (LLE).

The K_d_ corresponds to the dissociation constant between a ligand and the protein. Its value indicates the bond strength between them [31,68]. The following equations calculate the K_d_ parameter:

(4) $$K_{d}\;=\;10^{\left[\frac{{\Delta G}_{\mathrm{docking}}}{2.303\mathrm{RT}}\right]}.$$

According to Equation (4), ΔG_docking_ is the binding energy (kcal/mol) obtained from docking experiments; R is the gas constant whose value is 0.001987 kcal/mol·K; and T is the temperature in Kelvin, 298.15 K in our case.

Ligand efficiency index (LE) is a measure of binding energy and the compound’s size [31,70]. This parameter compares molecules according to their average binding energy and can be calculated according to the following equation [31]:

(5) $$\mathrm{LE}\;=\;\frac{-2.303\mathrm{RT}}{\mathrm{HAC}}\log\left(K_{d}\right)$$

In Equation (5), K_d_ is the dissociation constant obtained from Equation (4), and HAC corresponds to the non-hydrogen atoms (heavy atom counter) number in a ligand.

Binding efficiency index (BEI) is a measure that involves a ligand-binding property with the protein against molecular weight [71,72]. In this work, the binding efficiency index was calculated through the following equation [32]:

(6) $$\mathrm{BEI}\;=\;\left[{\mathrm{pK}}_{d}\mathrm{MW}\right]$$

where pK_d_ is −LogK_d_ and K_d_ is the dissociation constant calculated from Equation (4). MW represents the molecular weight in kDa.

Lipophilic ligand efficiency (LLE) has been defined as the difference between the ligand activity and lipophilicity (cLogP) and is obtained from the following Equation (7) [73].

(7) $${\mathrm{LLE}\;=\;\mathrm{pK}}_{d}-\mathrm{cLog}\left(P\right)$$

In Equation (7), pK_d_ is −LogK_d_ and cLogP is a ligand lipophility measure, calculated using the SwissADME web server [39].

### 3.5. ADME-Tox Properties

The absorption, distribution, metabolism, and excretion (ADME) properties of all ligands were calculated from optimized geometry of the molecules under study by using the SwissADME web server [39]. Other physicochemical variables like molecular weight (MW), octanol/water partition coefficient (cLogP), hydrogen bond acceptor (HBA), hydrogen bond donor (HBD), topological polar surface area (TPSA), and rotatable bond count (RB), respectively, were also calculated as the fundamental goal was to obtain ties of a compound to become a drug. We could predict the drug’s toxicological properties (Tox) considering the Lipinski [40,74], Veber [41], and Pfizer toxicity empirical rules [42] (Table 5).

## 4. Conclusions

Bacterial diseases have increased worldwide. This type of disease is caused by a large group of Gram-Positive and Gram-Negative bacteria. Several factors influence the virulence of these bacteria. It has been suggested that one of the virulence factors correspond to the widely studied proteins of the M4 family metalloproteins, with Zn^2+^ ion in their active center. In previous works, we designed a series of thermolysin inhibitor ligands (M4 family metalloproteinase). To determine if these designed compounds would be good antibacterial agents, we successfully constructed and applied a computational protocol with a complete analysis result obtained from docking experiments, molecular dynamics simulations, MM-PBSA, ligand efficiency calculation, and ADME-Tox prediction.

We concluded that compounds Lig783, Lig2177, and Lig3444 showed the best performance in the docking experiments. These same compounds also had very similar molecular dynamics and MM-PBSA behavior. These results agreed with the ligand efficiency analysis, demonstrating that these molecules can be suggested as good antibacterial agents. However, the results from the ADME-Tox properties analysis suggests that the unique candidate to be employed as an antibacterial agent amongst the designed ligands is Lig783.

## Figures and Tables

**Table 1:** Calculated docking ΔG_binding_ energies (kcal/mol) and root-mean-square deviation (RMSD) (Å) of the firsts ranked Autodock Vina poses of ligand-thermolysin complexes.

	Docking Pose-1		Docking Pose-2		Docking Pose-3	
	ΔG_binding_	RMSD	ΔG_binding_	RMSD	ΔG_binding_	RMSD
Lig5H9-5DPF ^1^	−8.2	0.94	−7.7	1.80	−7.5	2.36
Lig783-5DPF	−8.0	1.07	−8.0	1.16	−7.8	1.80
Lig1022-5DPF	−7.9	0.90	−7.8	1.88	−7.7	1.46
Lig1392-5DPF	−7.3	3.02	−7.2	4.02	−6.8	5.06
Lig2177-5DPF	−8.0	2.45	−7.9	3.01	−7.8	3.76
Lig3444-5DPF	−8.1	1.16	−8.0	4.18	−7.9	4.57
Lig6199-5DPF	−7.0	6.76	−6.9	6.81	−6.6	6.85

^1^ 5H9 (*N*-[(*S*)-([(benzyloxy) carbonyl]amino-methyl)(hydroxy)phosphoryl]-l-leucyl-4-methyl-l-leucine) was used as the reference molecule, and was re-docked using the same docking procedure employed for the other ligands.

**Table 2:** Average values and standard deviation of parameters taken from 50 ns of trajectories.

Complexes	RMSD (Å)	Number of H-Bond	Rg (Å)
Lig5H9-5DPF ^1^	1.11 ± 0.13	1.48 ± 1.68	5.36 ± 0.52
Lig783-5DPF	0.93 ± 0.08	0.17 ± 0.54	3.56 ± 0.02
Lig1022-5DPF	1.02 ± 0.11	0.09 ± 0.32	5.88 ± 0.86
Lig1392-5DPF	1.10 ± 0.15	0.35 ± 0.82	4.25 ± 0.04
Lig2177-5DPF	0.90 ± 0.07	0.46 ± 0.73	5.78 ± 0.29
Lig3444-5DPF	0.98 ± 0.08	0.74 ± 1.26	3.87 ± 0.03
Lig6199-5DPF	0.97 ± 0.08	0.05 ± 0.26	4.99 ± 0.14

^1^ 5H9 (*N*-[(*S*)-([(benzyloxy) carbonyl]amino methyl)(hydroxy)phosphoryl]-l-leucyl-4-methyl-l-leucine) was our reference molecule and was re-docked using the same docking procedure employed for the rest of the ligands.

**Table 3:** Predicted relative molecular mechanics Poisson-Boltzmann surface area (MM-PBSA) for the free energies (kcal/mol) and individual energy contributions of the complexes taken from 50 ns of trajectories.

Complexes	∆G_binding_	∆E_elect_	∆E_vdw_	∆G_polar_	∆G_Apolar_
Lig5H9-5DPF ^1^	−146.79 ± 8.30	−150.45 ± 13.06	−227.00 ± 7.61	254.72 ± 9.29	−24.05 ± 0.74
Lig783-5DPF	−60.84 ± 11.32	−24.72 ± 14.56	−81.13 ± 0.29	55.27 ± 17.78	−10.26 ± 0,84
Lig1022-5DPF	−114.11 ± 25.88	−1.84 ± 5.19	−120.97 ± 20.03	25.89 ± 25.87	−17.12 ± 2.35
Lig1392-5DPF	−75.79 ± 11.25	−23.69 ± 6.66	−87.35 ± 8.27	46.35 ± 12.71	−11.09 ± 1.40
Lig2177-5DPF	−37.06 ± 10.44	−42.08 ± 10.52	−63.36 ± 15.97	78.06 ± 17.56	−9.07 ± 2.28
Lig3444-5DPF	−27.31 ± 11.72	−7.94 ± 6.26	−49.88 ± 20.82	36.92 ± 21.27	−6.41 ± 2.67
Lig6199-5DPF	−88.56 ± 19.45	−74.95 ± 15.19	−159.04 ± 16.52	165.67 ± 33.93	−20.23 ± 1.22

^1^ 5H9 (*N*-[(*S*)-([(benzyloxy) carbonyl]amino methyl)(hydroxy)phosphoryl]-l-leucyl-4-methyl-l-leucine) was our reference molecule and was re-docked with the same docking procedure of the ligands.

**Table 4:** Ligand efficiency parameters calculated and absorption, distribution, metabolism, excretion, and toxicity (ADME-Tox) properties prediction for all molecules studied.

Ligands	MW (kDa)	K_d_	clogP	LE	BEI	LLE	HBA	HBD	TPSA (Å^2^)	RB
Lig5H9 ^1^	0.4855	9.78 × 10^−7^	2.71	0.248	12.37	3.29	8	5	163.87	16
Lig783	0.2723	1.37 × 10^−6^	2.60	0.400	21.52	3.26	2	2	40.46	0
Lig1022	0.4507	1.62 × 10^−6^	5.86	0.239	12.84	−0.07	2	0	34.14	14
Lig1392	0.3574	4.46 × 10^−6^	2.81	0.280	14.96	2.54	3	3	74.35	5
Lig2177	0.4805	1.37 × 10^−6^	3.45	0.235	12.19	2.41	5	2	114.67	0
Lig3444	0.3561	1.15 × 10^−6^	2.31	0.368	16.66	3.62	3	3	74.72	0
Lig6199	0.5106	7.64 × 10^−6^	4.06	0.189	10.04	1.07	7	1	88.56	13

^1^ 5H9 (*N*-[(*S*)-([(benzyloxy) carbonyl]amino methyl)(hydroxy)phosphoryl]-l-leucyl-4-methyl-l-leucine) was our reference molecule and was re-docked with the same docking procedure employed for the rest of ligands.

**Table 5:** Empirical rules for predicting the oral availability and toxicity properties of the studied ligands.

Properties	Oral Availability		Toxicity
	Lipinski Rules	Veber Rules	Pfizer 3/75 Rules
MW	≤500	-	-
cLogP	≤5	-	≤3
HBA	≤10	-	-
HBD	≤5	-	-
TPSA	-	≤140	≤75
RB	-	≤10	-

MW: Molecular weight; LogP: Octanol/water partition coefficient; HBA: Hydrogen bond acceptor; HBD: Hydrogen bond donor; TPSA: Topological polar surface area; RB: Rotatable bond count.

## Data Availability

Data available in a publicly accessible repository.

## Sample Availability

Samples of the compounds are available from the authors.
